# Communication between cancer patients, caregivers and oncologists about out-of-pocket spending

**DOI:** 10.1093/eurpub/ckac130.012

**Published:** 2022-10-25

**Authors:** A Tur-Sinai, N Bentur, D Urban

**Affiliations:** 1 Health Systems Management, The Max Stern Yezreel Valley College, Yezreel Valley, Israel; 2 School of Health Professions, Tel-Aviv University, Tel-Aviv, Israel; 3 Oncology, Sheba Medical Center, Tel Hashomer, Israel

## Abstract

**Background:**

Concern about the cost and affordability of cancer drugs is widespread and well known. Even in countries with universal healthcare systems or health insurance for all, additional patient out-of-pocket (OOP) expenses are prevalent. Studies showed that honest communication between oncologists and patients is an important component in alleviating financial burden of cancer care. The study explores patient-caregiver-oncologist communication regarding the affordability of OOP medication and the extent to which this communication is related to families’ financial burden after their loved ones’ death.

**Methods:**

A cross-sectional survey is conducted in Israel among 491 primary caregivers of deceased cancer patients, Jewish and Arab, in three oncology centers.

**Results:**

About 43% caregivers said that they and/or the patients had paid OOP for medications during the last half-year of the patient's life. Most (73%) oncologists who suggested an OOP medication hardly asked or did not ask about financial ability and took little or no interest in ability to afford it, 43% hardly explained or did not explain the advantages of an OOP medication, and 52% hardly explained or did not explain any treatment alternatives. A linear regression analysis reveals that older age and female gender are related to less communication about an OOP medication and that better education, greater affluence, and having private health insurance are related to more communication. About 56% of caregivers say that OOP payment for medications inflicted a very heavy or heavy financial burden on patients and their households. A regression analysis revealed that physicians’ interest in their financial ability and in the explanation they gave decreased their burden.

**Conclusions:**

Discussing and explaining the meaning of OOP payment alleviates the financial burden that families experience. It is crucial to develop and invest in improving oncologists’ education and skills to communicate costs more openly.

**Key messages:**

- Few oncologists took much interest in patients’ ability to afford medications, while most caregivers found OOP payment for medications burdensome.
- Oncologists’ explanations and interest in patients’ means lightened the burden. Oncologists need to be better educated in speaking about costs.

